# Identification and verification of a prognostic autophagy-related gene signature in hepatocellular carcinoma

**DOI:** 10.1038/s41598-024-53565-4

**Published:** 2024-02-06

**Authors:** Zhen Ma, Mali Chen, XiaoLong Liu, Hongbin Cui

**Affiliations:** 1 The Second Clinical Medical School, Lanzhou University, No. 82, Cuiyingmen, Chengguan District, Lanzhou City, 730000 Gansu Province China; 2 The Second Hospital of Lanzhou University, Lanzhou, 730030 Gansu China; 3 Key Laboratory of the Digestive System Tumors of Gansu Province, Lanzhou, 730030 China; 4 Department of Obstetrics, Gansu Provincial Maternity and Child-Care Hospital, 143 North Street, Qilihe District, Lanzhou City, 730030 Gansu Province People’s Republic of China

**Keywords:** Autophagy-related genes, HCC, Immunity, Immune checkpoint, Prognosis, Cancer genetics, Clinical genetics

## Abstract

This study aimed to investigate the potential of autophagy-related genes (ATGs) as a prognostic signature for HCC and explore their relationships with immune cells and immune checkpoint molecules. A total of 483 samples were collected from the GEO database (n = 115) and The Cancer Genome Atlas (TCGA) database (n = 368). The GEO dataset was used as the training set, while the TCGA dataset was used for validation. The list of ATGs was obtained from the human autophagy database (HADB). Using Cox regression and LASSO regression methods, a prognostic signature based on ATGs was established. The independent use of this prognostic signature was tested through subgroup analysis. Additionally, the predictive value of this signature for immune-related profiles was explored. Following selection through univariate Cox regression analysis and iterative LASSO Cox analysis, a total of 11 ATGs were used in the GEO dataset to establish a prognostic signature that stratified patients into high- and low-risk groups based on survival. The robustness of this prognostic signature was validated using an external dataset. This signature remained a prognostic factor even in subgroups with different clinical features. Analysis of immune profiles revealed that patients in the high-risk group exhibited immunosuppressive states characterized by lower immune scores and ESTIMATE scores, greater tumour purity, and increased expression of immune checkpoint molecules. Furthermore, this signature was found to be correlated with the infiltration of different immune cell subpopulations. The results suggest that the ATG-based signature can be utilized to evaluate the prognosis of HCC patients and predict the immune status within the tumour microenvironment (TME). However, it is important to note that this study represents a preliminary attempt to use ATGs as prognostic indicators for HCC, and further validation is necessary to determine the predictive power of this signature.

## Introduction

Liver cancer is a prevalent and highly malignant disease. Hepatocellular carcinoma (HCC) is the most common form of liver cancer and accounts for 75–85% of cases of liver cancer^1^. The worldwide five-year survival rate for patients with HCC is only 18%, with even lower rates observed in many Asian countries^2^. Consequently, HCC imposes a substantial disease burden on both individual families and society as a whole.

Identifying prognostic predictors for HCC patients holds immense significance. Currently, several factors, including AFP, ALT, and AST levels and the Child‒Pugh score, have been reported to be associated with patient prognosis. However, the use of individual factors alone is often insufficient for accurately predicting patient outcomes due to the significant heterogeneity among cancer patients. The emergence of high-throughput sequencing technology has led to the discovery of novel prognostic factors for cancer patients at the genetic level.

Autophagy is a highly dynamic multi-step process regulated by many functional protein units^3^. Deng et al. collected more than 4000 regulatory proteins in autophagy and cell death pathways and found that these proteins and signaling pathways are significantly related to human diseases, and that includes tumors^4,5^. Autophagy-related proteins and pathways in hepatocytes play vital roles in maintaining liver homeostasis and preventing the onset of hepatocellular carcinoma. Furthermore, autophagy is involved in the cancer immune response^6^. Several studies have investigated the association between HCC and autophagy, with autophagy-related genes identified as potential therapeutic targets and prognostic factors for HCC^7,8^.

In this study, our objective was to develop and validate a prognostic signature for HCC using autophagy-related genes. Additionally, we aimed to investigate the association between this gene signature and immune-related profiles. By doing so, we sought to gain a deeper understanding of the impact of autophagy on cancer characteristics and potentially identify new strategies for cancer immunotherapies.

## Materials and methods

### Data collection and screening of prognostic ATGs

We obtained the HCC dataset GSE76427^9^, which consists of gene expression and clinical data from the GEO (https://www.ncbi.nlm.nih.gov/geo/), as our training set. This dataset was based on the gpl10558 (Illumina humanHT-12v4.0 expression beadchip) platform. Samples without survival information were excluded, resulting in the final inclusion of 115 liver cancer samples. For our validation set, we utilized TCGA (https://portal.gdc.cancer.gov/) data, which included expression profile and clinical data. We excluded samples without survival information, resulting in 368 liver cancer samples. The autophagy gene list was obtained from the Human Autophagy Database (HADB, http://autophagy.lu/clustering/index.html) and compiled into an ATG expression matrix. We performed univariate Cox analysis to select prognostic ATGs in the training set, with P < 0.05 considered to indicate statistical significance.

### GO and KEGG analyses of prognostic ATGs

Gene Ontology (GO) analysis is a widely utilized method for gene function annotation^10^. The analysis of pathway enrichment using the Kyoto Encyclopedia of Genes and Genomes (KEGG) is another common approach^11^. To investigate the importance of prognostic autophagy genes in HCC development, we conducted GO and KEGG analyses of these genes using the R clusterProfiler package. These analyses provide insights into the functional roles and pathways associated with the identified prognostic autophagy genes.

### ATG-based prognostic signature construction

In this study, machine learning was performed using the LASSO regression method to select variables based on a penalty function. The goal was to identify the optimal gene signature with the highest AUC value. LASSO regression was iteratively performed 1000 times for candidate genes. The resulting genetic combinations were evaluated, and the gene signature with the highest AUC was selected^12^. Subsequently, a risk score was calculated for each patient based on the expression levels of the relevant autophagy-related genes (ATGs). Patients were then categorized into high-risk and low-risk groups based on the median risk score. Cox regression analysis was employed to validate the prognostic gene signature based on the selected ATGs.

### External validation of the gene signature

In the independent external dataset, the risk score for each sample was calculated based on the established gene signature. Patients were divided into high-risk and low-risk groups according to the median risk score. Cox regression analysis was also conducted to further validate the prognostic significance of the gene signature. The survival of HCC patients in the high-risk and low-risk groups was evaluated using the Kaplan‒Meier method. Additionally, the prognostic performance of the optimal gene signature in the validation dataset was assessed using receiver operating characteristic (ROC) curves. This allowed for an evaluation of the predictive accuracy of the gene signature in terms of patient outcomes.

### Pathway and mechanism exploration for the gene signature

To investigate the important pathways enriched in the different risk groups, we performed GSEA (gene set enrichment analysis) and GSVA (gene set variation analysis). For the GSEA, we utilized GSEA software (version 4.0.3) and used "h.all.v7.1.symbols.gmt" and "c7.all.v7.1.symbols.gmt" as reference gene sets. We conducted 1000 genome replacements to obtain a standardized enrichment score for each analysis. A nominal p value of < 0.05 and a false discovery rate of < 0.05 were considered significant. With "h.all.v7.1.symbols.gmt" as the reference gene set, we performed GSVA using the clusterProfiler package and GSVA package. An adjusted p value of < 0.05 was considered to indicate statistical significance. These analyses allowed us to identify important pathways that were enriched in different risk groups based on the established gene signature.

### Immune mechanism exploration according to the signature

With the transcriptome expression profile data, we calculated the interstitial score, immune score, ESTIMATE score, and tumour purity using the ESTIMATE algorithm to investigate the differences in immune molecules between two distinct groups: the high-risk group and the low-risk group. To assess the composition of 22 types of immune cells in both groups, we utilized the CIBERSORT web tool based on linear support vector regression and the deconvolution method^13^.

The filtered data were subjected to principal component analysis (PCA) to detect variations in immune cell infiltration, allowing for the creation of a two-dimensional PCA clustering graph using the ggplot2 R package. The correlations, infiltration differences, and interactions of the 22 immune cell types were visualized using the corrplot^14^, ggplot2^15^, and iGraph packages^16^.

Furthermore, we generated Kaplan‒Meier curves to explore the relationship between immune cell composition and patient prognosis. To investigate the potential correlation between an optimal prognostic gene signature and immune cell infiltration, we performed correlation analysis and visualized the results using the ggplot2 package.

Overall, this approach provides valuable insights into the immune microenvironment of high-risk and low-risk patients, contributing to the understanding of the underlying mechanisms and potential therapeutic targets in the context of cancer immunotherapy. These findings have the potential to inform future research and clinical practice.

### Correlation analysis between the signature and immune checkpoint molecules

To investigate the correlation between the prognostic gene signature and immune checkpoint molecules, we compared the expression levels of common immune checkpoint molecules, including CD27, CD40, CD70, TNFRSF14, CD276, VTCN1, IDO1, PDCD1, CD274, PDCD1LG2, HAVCR2, TIGIT, CTLA4, CD86, ICOS, LAG3, and CD58, between the high-risk and low-risk groups.

## Results

### GO and KEGG analysis

**Figure 1:**
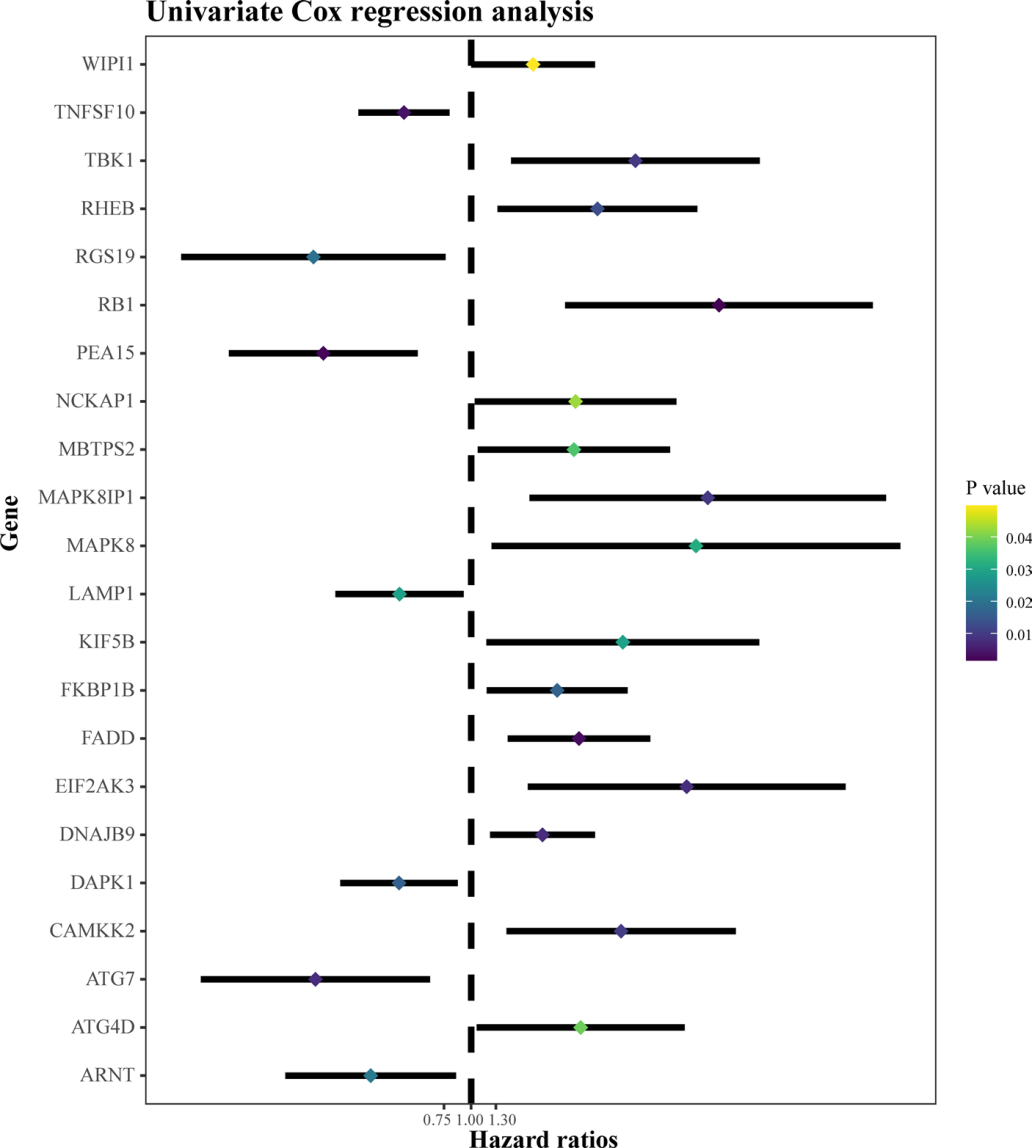
Selection of prognostic autophagy genes.

**Figure 2:**
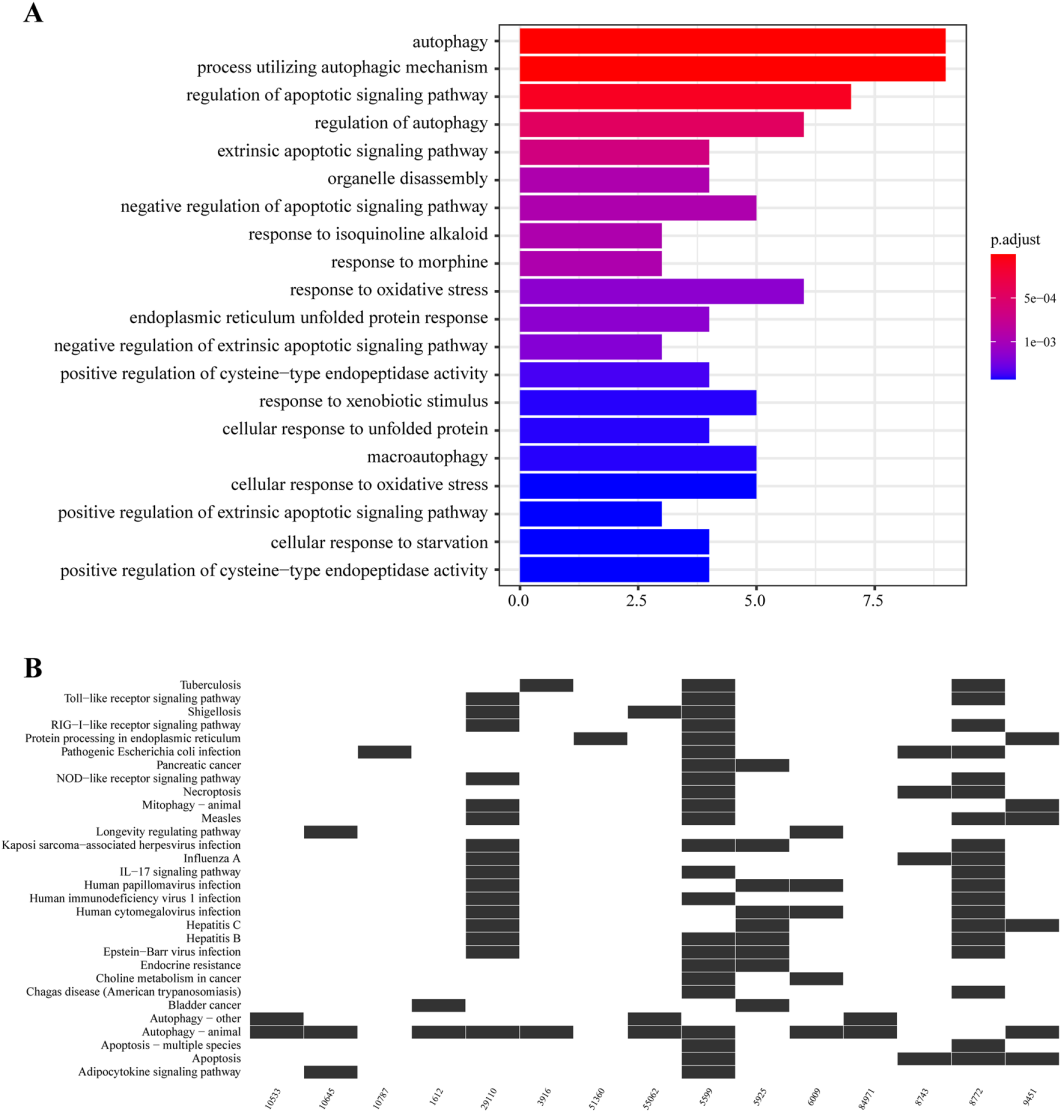
Results of GO and KEGG analyses of prognostic autophagy genes. (**A**) Results of GO analysis. The X-axis represents the number of genes enriched in a GO term, and the Y-axis represents the GO term. (**B**) Results of KEGG analysis. The X-axis represents the enriched genes, and the Y-axis represents the name of each enrichment pathway.

Using the Human Autophagy Database, we identified a total of 222 autophagy genes (ATGs). By performing univariate Cox regression analysis, we selected 22 prognostic ATGs (Fig. 1). Gene Ontology (GO) analysis was also conducted to explore the biological functions associated with these 22 prognostic ATGs. The analysis revealed that these genes are involved primarily in autophagy, the utilization of autophagic mechanisms, the regulation of apoptotic signalling pathways, the regulation of autophagy, extrinsic apoptotic signalling pathways, organelle disassembly, and the negative regulation of apoptotic signalling pathways (Fig. 2A).

Furthermore, KEGG analysis was performed to investigate the pathways in which these genes were enriched. The analysis demonstrated that these genes are mainly associated with the Toll-like receptor signalling pathway, RIG-I receptor-like signalling pathway, NOD-like receptor signalling pathway, and IL-17 signalling pathway (Fig. 2B).

### Construction of the ATG-based signature

**Figure 3:**
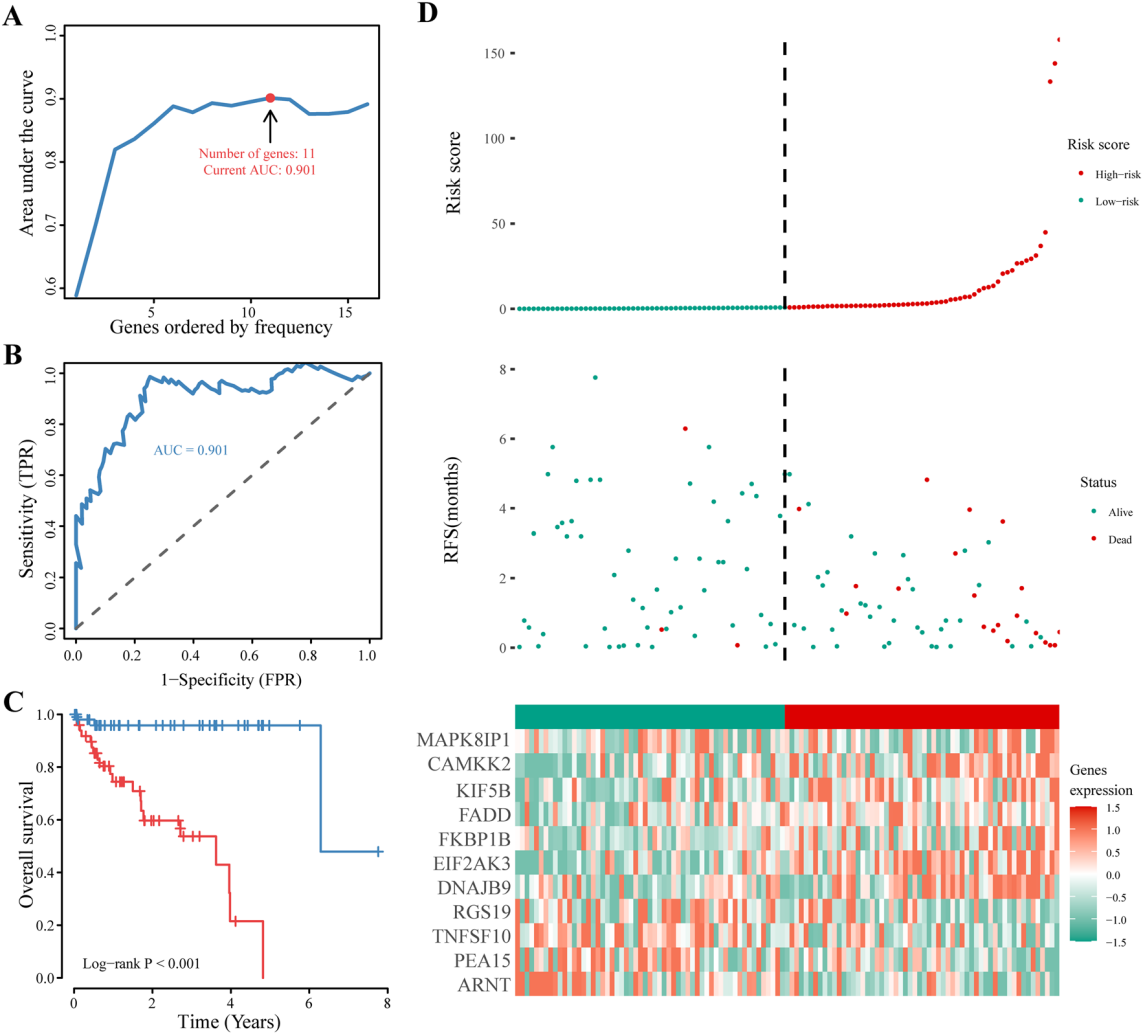
Construction of an ATG-based prognostic signature. (**A**) Iterative LASSO Cox regression analysis was used to identify the ATG-based signature. (**B**) ROC analysis for assessment of prognostic performance. (**C**) Kaplan–Meier curves of patients in both the low-risk group and high-risk group. (**D**) Risk plot, from top to bottom, representing the risk score distribution, survival status of each patient and expression heatmap of the signature genes in the low-risk group and high-risk group.

To achieve accurate prognosis prediction for hepatocellular carcinoma (HCC), we developed an optimal prognostic signature consisting of 11 autophagy genes (ATGs) using iterative LASSO Cox regression analysis (Fig. 3A). The performance of the signature was assessed by analysing the area under the receiver operating characteristic curve (AUC). The signature demonstrated good prognostic power in HCC patients, with an AUC of 0.901 (Fig. 3B).

Survival analysis revealed that patients in the high-risk group had significantly worse overall survival than did those in the low-risk group (p < 0.001; Fig. 3C). The risk plot shows the distribution of risk scores, survival status of each patient, and expression of genes included in the signature (Fig. 3D). The number of patient deaths in the high-risk group was significantly greater than that in the low-risk group, and the expression levels of each gene included in the gene signature were significantly different between the high-risk and low-risk groups.

Collectively, these results indicate that the established gene signature can accurately distinguish patients at different risk levels and demonstrate the significant prognostic value of this model for patients with liver cancer. This signature provides a potential tool for clinicians to identify high-risk patients and develop personalized treatment plans.

### Validation of the optimal ATG-based signature in an external dataset

**Figure 4:**
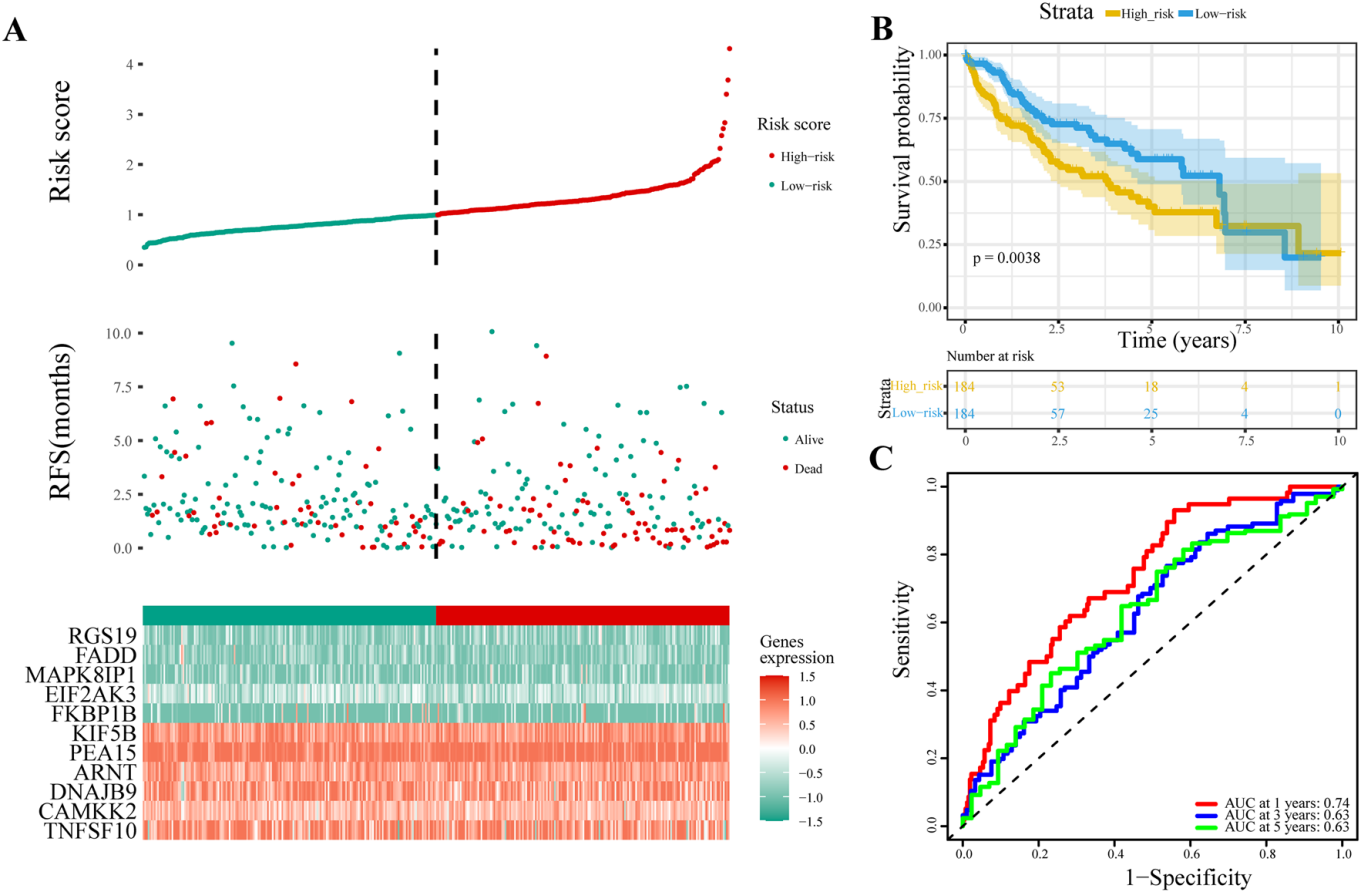
Validation of the ATG-based prognostic signature in an external dataset. (**A**) Risk plot of the external dataset. The data are presented from top to bottom for the risk score distribution, survival status of each patient and expression heatmap of the signature genes in the low-risk group and high-risk group. (**B**) Kaplan‒Meier curve of the external dataset. (**C**) ROC analysis of the external dataset.

To assess the robustness of the 11-gene signature model, we included an external dataset for further validation. The risk plot (Fig. 4A) demonstrated that the number of patient deaths in the high-risk group was significantly greater than that in the low-risk group, indicating the effectiveness of the signature in predicting patient outcomes.

Survival analysis using the external liver cancer dataset confirmed that the 11-gene signature could still successfully distinguish patients with different prognoses (p = 0.0038; Fig. 4B). This finding suggested that the predictive power of the signature extends beyond the original dataset, reinforcing its clinical relevance.

We performed ROC analysis to evaluate the prognostic ability of the 11-gene signature. The results indicated that the signature displayed good predictive accuracy for 1-year, 3-year, and 5-year survival rates (Fig. 4C).

### The independent use of the ATG-based signature for survival prediction in different clinicopathological subgroups

In HCC, age, BCLC stage, and TNM stage are recognized as important prognostic factors that can influence patient outcomes. To assess whether the 11-gene signature model functions independently of these clinicopathological factors, we performed survival analysis by regrouping patients based on different clinicopathological characteristics.

**Figure 5:**
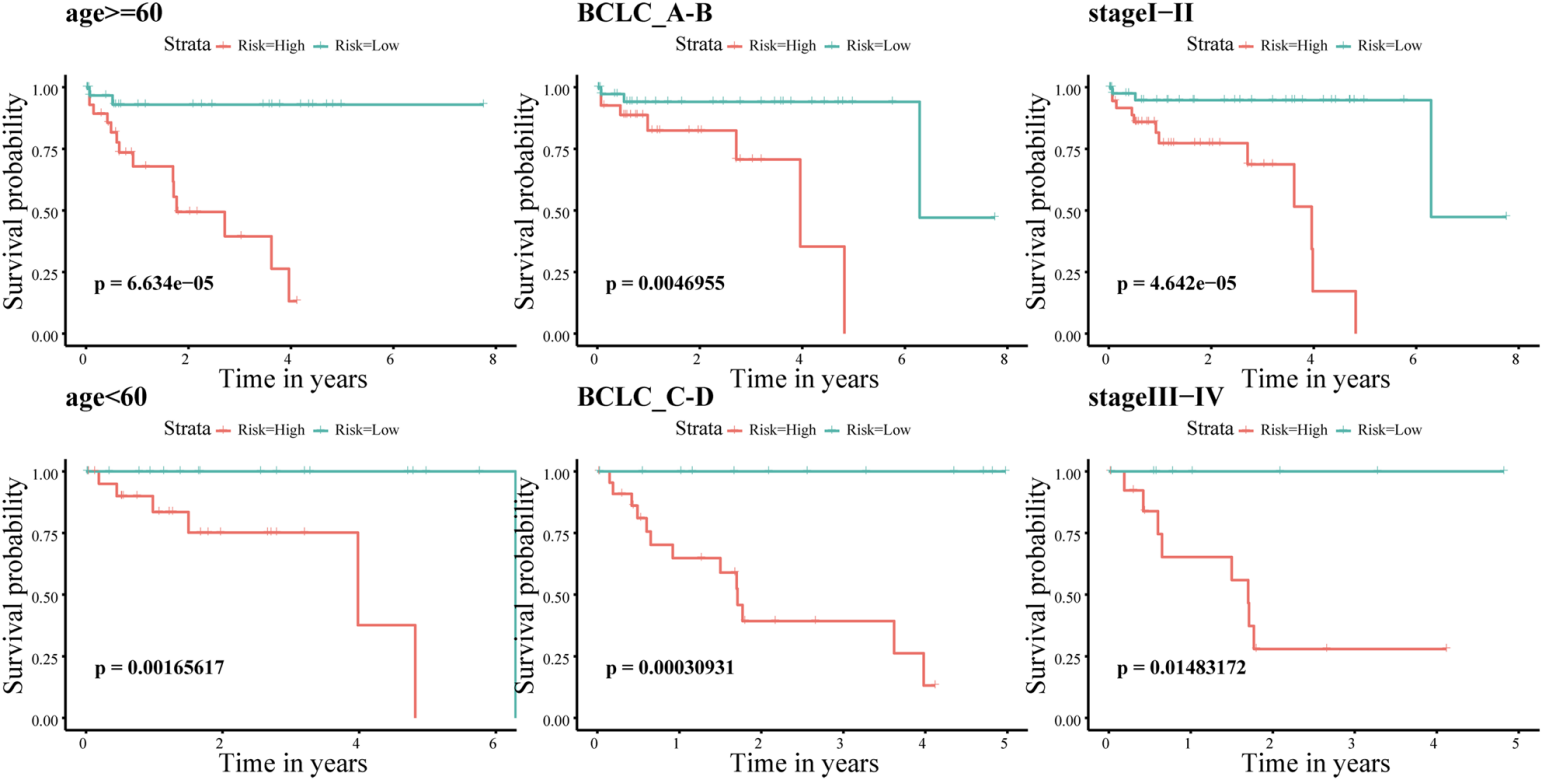
Kaplan–Meier curves for HCC patients in different clinical subgroups (divided according to age, BCLC stage, and TNM stage). The x-axis is the survival time (years); the y-axis is the survival probability. High-risk groups are presented in red, and low-risk groups are presented in blue.

The Kaplan‒Meier curves revealed that even after adjusting for clinical features, the survival of patients in the high-risk group remained consistently poorer than that of patients in the low-risk group (all p values < 0.05, as depicted in Fig. 5). These findings suggest that the 11-gene signature model retains its prognostic value for HCC patients regardless of age, BCLC stage, or TNM stage.

### Results of GSEA and GSVA

**Figure 6:**
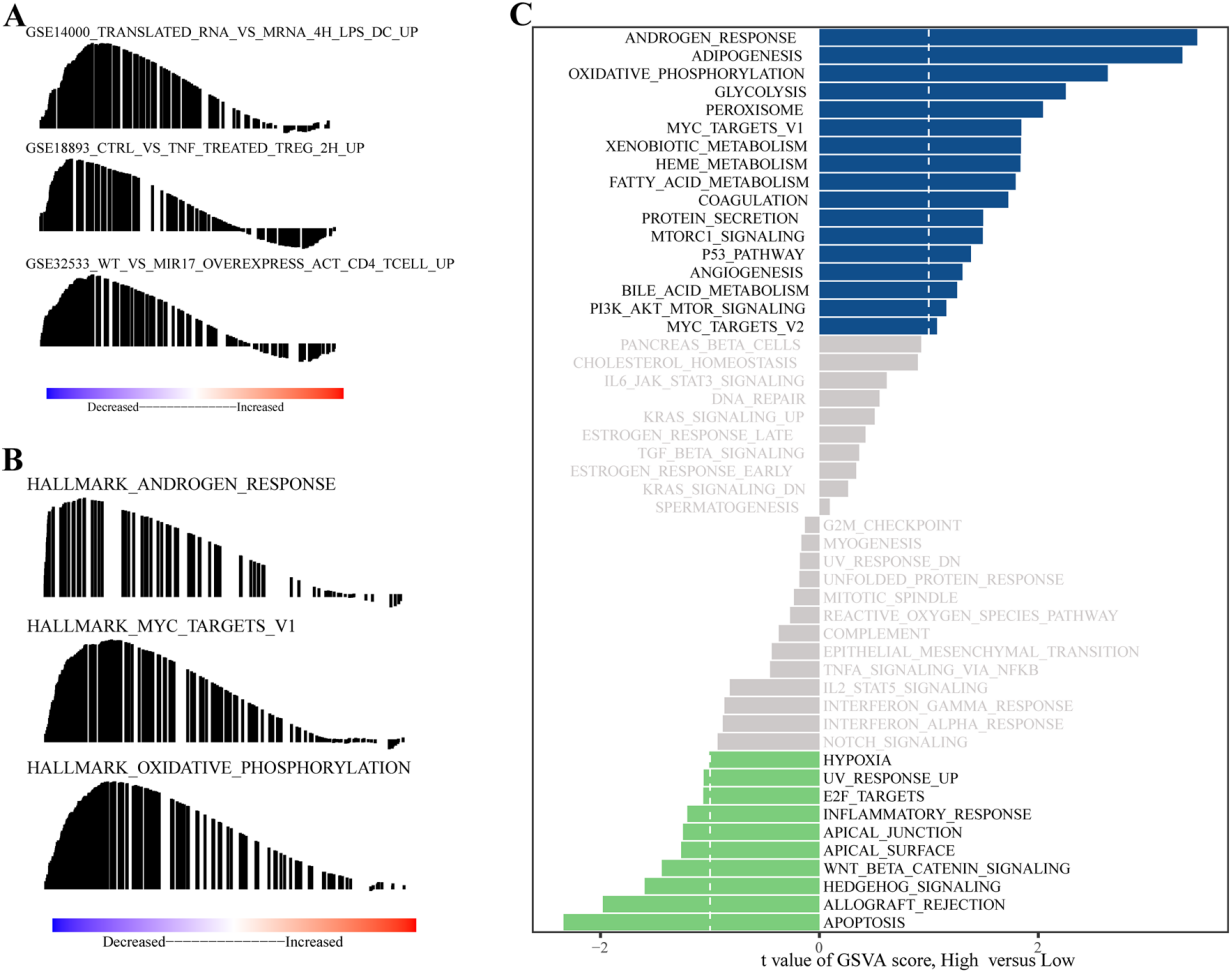
Results of GSEA and GSVA. (**A**) GSEA using the reference gene set of ‘h.all.v7.1.symbols.gmt’. (**B**) GSEA using the reference gene set ‘c7.all.v7.1.symbols.gmt’. (**C**) GSVA using the reference gene set of ‘h.all.v7.0.symbols.gmt’.

Gene set enrichment analysis (GSEA) and gene set variation analysis (GSVA) are indispensable tools for exploring significant differences in crucial functional phenotypes between high- and low-risk patients. In our study, we employed GSEA to identify enriched pathways specifically associated with the high-risk group, as presented in Fig. 6A and B.

The pathways significantly enriched in the high-risk group included the following:

- TRANSLATED_RNA_VS_MRNA_4H_LPS_DC
- CTRL_VS_TNF_TREATED_TREG_2H
- WT_VS_MIR17_OVEREXPRESS_ACT_CD4_TCELL
- ANDROGEN_RESPONSE
- MYC_TARGETS_V1
- OXIDATIVE_PHOSPHORYLATION

Moreover, our GSVA corroborated the activation of specific pathways within the high-risk group, as illustrated in Fig. 6C. The pathways activated in this group included the following:

- ANDROGEN_RESPONSE
- MYC_TARGETS_V1
- P53_PATHWAY
- PI3K_AKT_MTOR_SIGNALING

### Differences in tumour purity, ESTIMATE score, immune score and stromal score between the high- and low-risk patients

**Figure 7:**
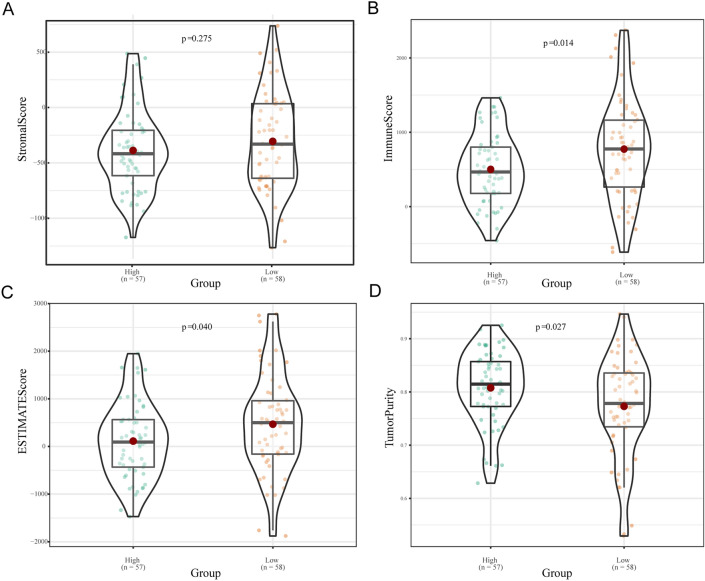
Differences in the stromal score, ESTIMATE score, tumour purity, and immune score between the high- and low-risk groups.

Comparisons of tumour purity, the ESTIMATE score, the immune score, and the stromal score between the high-risk and low-risk groups were conducted to investigate the underlying biological mechanisms associated with the 11-gene signature model. Our results showed that there was no statistically significant difference in the stromal score between the two groups, as demonstrated in Fig. 7A.

However, lower immune activity was observed in the high-risk group than in the low-risk group, as indicated by a significantly lower immune score (p = 0.014; Fig. 7B). Similarly, the ESTIMATE score was significantly lower in the high-risk group (p = 0.04; Fig. 7C).

In addition, the tumour purity of the high-risk group was significantly greater than that of the low-risk group (p = 0.027; Fig. 7D). This finding suggests that the high-risk group had a greater proportion of tumour cells than nontumor cells.

### Relationships between the signature and patient prognosis and immune infiltration status

**Figure 8:**
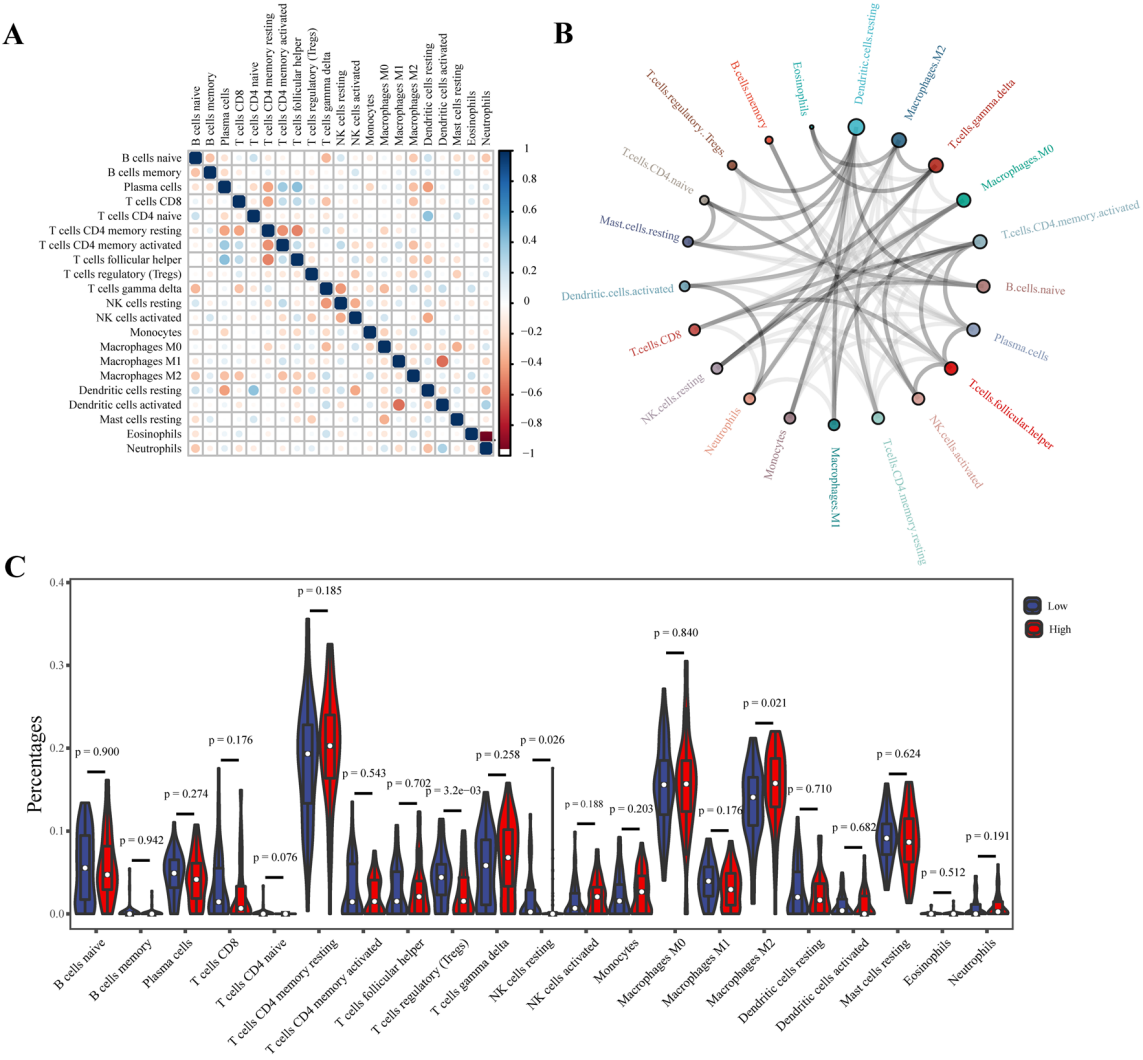
Analysis of immune cell infiltration. (**A**) Correlations among infiltrated immune cells. (**B**) Immune cell interaction network, with the circle from large to small indicating the intensity of the interaction from strong to weak. (**C**) Differences in immune cell infiltration between the high- and low-risk groups.

To investigate the differences in immune cell infiltration patterns between the high-risk and low-risk groups, an analysis of immune cell correlations was conducted. The results revealed several correlations among different immune cell types. Specifically, plasma cells were positively correlated with the infiltration of activated memory CD4 T cells and follicular helper T cells but negatively correlated with the infiltration of resting CD4 memory T cells and resting dendritic cells. Additionally, resting CD4 memory T cells exhibited a negative correlation with the infiltration of activated memory CD4 T cells (Fig. 8A).

Furthermore, an immune cell interaction network was constructed (Fig. 8B), which indicated that resting dendritic cells, M2 macrophages, and gamma delta T cells displayed the strongest interactions with other immune cells. However, eosinophils, memory B cells, and regulatory T cells (Tregs) exhibited weakened interactions with other cells. Analysis of the immune cell composition demonstrated that the infiltration of regulatory T cells (Tregs) and M2 macrophages was lower in the high-risk group (Fig. 8C).

**Figure 9:**
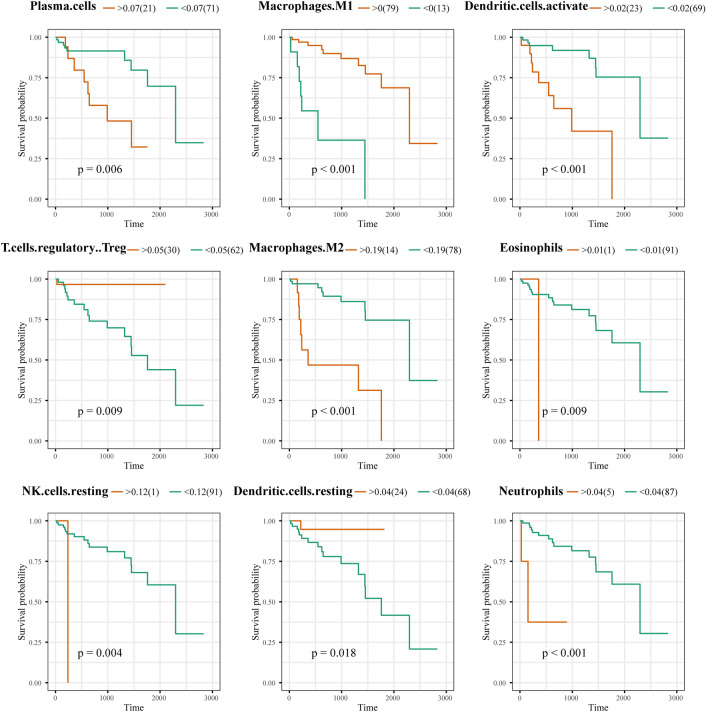
Survival analysis according to immune cell infiltration status. The x-axis represents the survival time (days). Orange represents the high infiltration group, and green represents the low infiltration group.

The relationship between immune cell infiltration and survival outcomes was also investigated (Fig. 9). The infiltration of immune cells, such as M1 macrophages (p < 0.001), regulatory Tregs (p = 0.009), and resting dendritic cells (p = 0.018), was associated with a favourable prognosis. Conversely, the infiltration of plasma cells (p = 0.006), activated dendritic cells (p < 0.001), M2 macrophages (p < 0.001), eosinophils (p = 0.009), resting NK cells (p = 0.004), and neutrophils (p < 0.001) was associated with a poorer prognosis.

**Figure 10:**
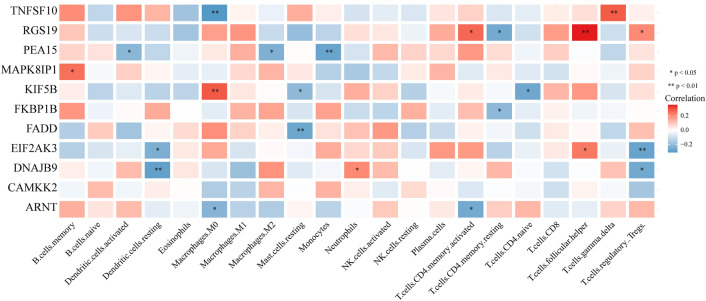
Correlation analysis between signature-included genes and prognostic immune cells.

To explore the relationship between the infiltration of immune cells and the signature genes, a correlation heatmap was constructed (Fig. 10). The analysis revealed that TNFSF10 was negatively correlated with the infiltration of M0 macrophages, while KIF5B was positively correlated with the infiltration of M0 macrophages. Additionally, RGS19 expression was positively correlated with the infiltration of CD4 memory-activated T cells, follicular helper T cells, and regulatory T cells but negatively correlated with the infiltration of resting CD4 memory T cells.

### Comparison of immune checkpoint molecule expression in the high- and low-risk groups

**Figure 11:**
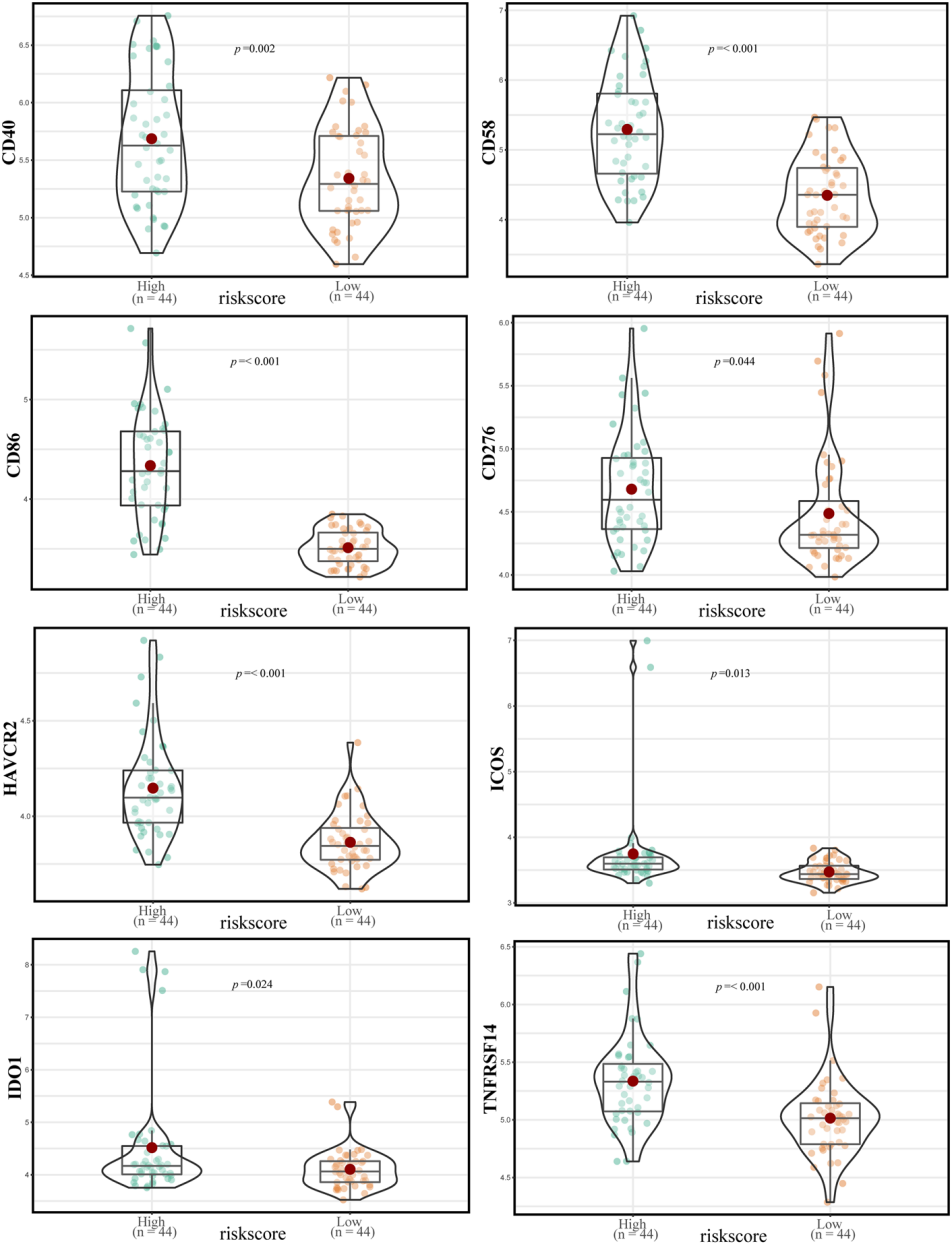
Comparison of immune checkpoint molecule expression between the high- and low-risk groups.

An analysis of immune checkpoint molecules in the high-risk and low-risk groups revealed significant differences in the expression levels of various immune checkpoint molecules (Fig. 11). Specifically, the expression levels of CD40 (p = 0.002), CD58 (p < 0.001), CD86 (p < 0.001), CD276 (p = 0.044), HAVCR2 (p < 0.001), ICOS (p = 0.013), IDO1 (p = 0.024), and TNFRSF14 (p < 0.001) were increased in the high-risk group compared to the low-risk group.

## Discussion

Due to the high incidence and mortality rates associated with HCC, a growing number of prognostic markers have been developed for HCC patients. Despite these efforts, reliable biomarkers based on gene expression profiles remain scarce. In this study, we employed the LASSO-Cox regression algorithm to develop a prognostic signature comprising 11 autophagy-related genes (MAPK8IP1, CAMKK2, KIF5B, FADD, FKBP1B, EIF2AK3, DNAJB9, RGS19, TNFSF10, PEA15, and ARNT) for HCC. MAPK8IP1 is a JNK-interacting protein that selectively mediates JNK signalling. Its role in liver cancer has not been fully studied. However, in gastric cancer, its expression is decreased, and overexpression of MAPK8IP1 can inhibit the metastatic ability of gastric cancer cells^17^. CAMKK2 is a serine/threonine protein kinase and belongs to the Ca^2+^/calmodulin-dependent protein kinase subfamily. Its expression is significantly upregulated in HCC and is negatively correlated with the prognosis of HCC patients^18^. KIF5B is a member of the kinesin motor-driven superfamily and plays an important role in basic cellular processes. KIF5B expression is upregulated in HCC cell lines and tissues. Silencing KIF5B can significantly reduce the proliferation, invasion, and metastasis of liver cancer cells^19^. Fas-associated protein with death domain (FADD) is a crucial adapter protein for cell apoptosis in the death receptor pathway. Thus, cells lacking FADD promote necrotic apoptosis^20^. FADD is inhibited in HCC, and its reduced expression inhibits its role in mediating liver cancer cell apoptosis^21^. FKBP1B is a member of the FKBP (FK506-binding protein) family and plays a critical role in plant growth and development^22^. This family of genes plays an inhibitory role in tumour progression, including the inhibitory effect of FKBP5 on breast cancer and the antiapoptotic effect of FKBP51 in melanoma^23,24^. However, the roles of these family members in HCC has not been well studied. EIF2AK3 (tumour-activating protein kinase R (PKR)-like endoplasmic reticulum kinase) is also known as PERK, and its inactivation leads to an increase in ferroptosis in colorectal cancer cells^25^. In renal clear cell carcinoma, inhibiting the expression of PERK can have an antitumour effect^26^. In HCC, PERK is upregulated and associated with resistance to sorafenib^27^. DNAJB9 is a member of the heat shock protein 40 family and plays a multifunctional role in maintaining client protein and cellular homeostasis. It inhibits the invasion, migration, and metastasis of cancer cells in triple-negative breast cancer^28^. Currently, we have not found any relevant studies on DNAJB9 or its role in HCC. The expression of regulator of G protein signalling 19 (RGS19), a regulator of the RGS family of G protein signalling pathways, is upregulated in many tumours, including HCC^29^. RGS19 is associated with carcinogenesis in several types of cancers, including ovarian cancer, gastric cancer, and prostate cancer^29–31^. Currently, there is limited research on the relationship between RGS19 and HCC. Tumour necrosis factor-related apoptosis-inducing ligand 10 (TNFSF10) is a death ligand cytokine that is mainly expressed by effector immune cells to kill malignantly transformed cells^32^. As an autophagy gene, TNFSF10 has the potential to predict the prognosis of melanoma patients and serve as a therapeutic marker for melanoma^33^. TNFSF10 is downregulated in HCC and may serve as a potential therapeutic target for HCC^34^. Proliferation and Apoptosis Adaptor Protein 15 (PEA15) consists of 130 amino acid residues and performs various antiapoptotic functions. It is also involved in the development of tumours, such as breast cancer, thyroid cancer, and colon cancer tumours^35^. In HCC, PEA15 has been found to promote HCC cell migration and increase HCC cell resistance to sorafenib^36^. The aryl hydrocarbon receptor translocator (ARNT) is a ligand-dependent transcription factor that regulates the adaptability and maladaptation to external and internal signals. ARNT is closely related to the pathogenesis of many diseases, including cancer^37^. In HCC, ARNT is considered a key factor in the pathogenicity of aflatoxins^38^.

With respect to the prognostic model based on the 11 autophagy-related genes (ATGs), we calculated risk scores for each HCC patient and stratified them into high- and low-risk groups using the median risk value as the cut-off. The Kaplan–Meier curve analysis demonstrated that patients in the high-risk group had significantly shorter survival than did those in the low-risk group, indicating the success of our model in predicting patient outcomes. We further validated the robustness of this signature in an independent test set and found that the model had high discriminatory power, as evidenced by the area under the ROC curve. Our ATG-based signature was identified as an independent risk factor for HCC patients and was associated with survival across different clinical subgroups.

ATGs have been demonstrated to be closely associated with cancer progression and patient prognosis in various cancer types, including breast, lung, colorectal, and bladder cancers^39–42^. Similarly, the prognostic value of autophagy has been reported in HCC patients, although the underlying mechanism remains poorly understood. To investigate the role of autophagy in tumour tissues, we conducted GSEA and GSVA. The results revealed a highly enriched and activated pathway known as the androgen response pathway in the high-risk group. The androgen response pathway has been found to have significant effects on the immune system and is capable of altering and suppressing immune responses^43^. These findings suggest that autophagy may modulate cancer characteristics by influencing antitumour immunity, ultimately impacting the prognosis of HCC patients.

To further elucidate the relationship between autophagy and the immune response, we employed several analytical methods related to immune profiling. These methods included tumour purity assessment, the ESTIMATE score, the immune score, and the score matrix. Our findings revealed that the immune score of the high-risk group was lower than that of the low-risk group. The immune score has been established as a superior predictor of cancer prognosis and treatment outcomes. Tumour samples often contain a mixture of cancer cells and normal cells in varying proportions, and the proportion of cancer cells within the tumour sample is referred to as tumour purity^44^. Tumour purity is an important confounding factor when evaluating the correlation between gene expression and clinicopathological features^45–47^. In our study, the tumour purity in the high-risk group was greater than that in the low-risk group. However, there was no statistically significant difference in the interstitial score between the two groups. These findings suggest that the immune response within the tumour microenvironment (TME) is more suppressed in high-risk patients than in low-risk patients. Furthermore, we analysed the relationship between immune cell infiltration and autophagy. However, the differences in immune cell profiles between the two groups did not align with the clinical effects of ATGs. This inconsistency may be attributed to the diverse types of immune cells and the influence of various confounding factors.

With an improved understanding of cancer immunity, immunotherapy, particularly immune checkpoint inhibitors, has achieved significant success in treating human cancers^48–51^. In the case of HCC, immunotherapy has emerged as a promising therapeutic option^52^. However, the response rate to immunotherapy is less than one-third of patients. The expression of immune checkpoint genes is often used as a biomarker for the potential benefit of immunotherapy. We compared the expression levels of several immune checkpoint genes between the high-risk and low-risk groups and found that the high-risk group exhibited significantly greater expression of these genes. This finding suggests that patients in the high-risk group may be more suitable candidates for immunotherapies based on immune checkpoint inhibitors.

Despite the effectiveness of our 11 ATG-based signature in determining patient prognosis and predicting patient response to immunotherapy, certain limitations still exist in our study. First, we used public retrospective datasets for our analysis, and further prospective validation in external datasets is needed. Second, while autophagy-related genes are important cancer-related genes, our analysis did not consider the effects of other genes. Therefore, the power of this prognostic signature may be limited. Third, the prediction of immunotherapeutic response was based on indirect evidence. Thus, it is crucial to test the predictive ability of this signature for immunotherapy efficacy in relevant cohorts.

## Conclusion

This study systematically investigated the expression profiles of ATGs in various HCC patients. We successfully developed and validated an 11-ATG-based prognostic model specifically for HCC patients. Additionally, this prognostic model also exhibited predictive ability for the immune status within the tumour microenvironment (TME) to some extent. The findings from this study offer a new prognostic determinant for HCC patients and have the potential to optimize the use of immunotherapy in HCC patients.

## Data Availability

All the data generated or analysed during this study are included in this manuscript.
